# Community Structures in Bipartite Networks: A Dual-Projection Approach

**DOI:** 10.1371/journal.pone.0097823

**Published:** 2014-05-16

**Authors:** David Melamed

**Affiliations:** Department of Sociology, University of South Carolina, Columbia, South Carolina, United States of America; Mälardalen University, Sweden

## Abstract

Identifying communities or clusters in networked systems has received much attention across the physical and social sciences. Most of this work focuses on single layer or one-mode networks, including social networks between people or hyperlinks between websites. Multilayer or multi-mode networks, such as affiliation networks linking people to organizations, receive much less attention in this literature. Common strategies for discovering the community structure of multi-mode networks identify the communities of each mode simultaneously. Here I show that this combined approach is ineffective at discovering community structures when there are an unequal number of communities between the modes of a multi-mode network. I propose a dual-projection alternative for detecting communities in multi-mode networks that overcomes this shortcoming. The evaluation of synthetic networks with known community structures reveals that the dual-projection approach outperforms the combined approach when there are a different number of communities in the various modes. At the same time, results show that the dual-projection approach is as effective as the combined strategy when the number of communities is the same between the modes.

## Introduction

Discovering the community structure of bipartite networks often entails either examining the community structure of one of the modes of the network, or determining the community structure of both modes simultaneously using a ‘combined’ approach. The former has been shown to result in a loss of substantial structural information [1], which in turn results in a loss of analysts’ ability to uncover the function and topology of the network as a whole. The latter may be inefficient at recovering the community structure of both modes because relations between nodes in one mode are inferred based on indirect ties through the second mode, and the computations for nodes in one of the modes are affected by the nodes in the other mode. As such, the present paper advocates a dual-projection approach to uncovering the community structure of bipartite networks.

Some instances of bipartite graphs include affiliation networks which link people to committees [2], or Petri nets which are used in computer science designs of concurrent systems [3]. The dual-projection approach to community detection in such bipartite networks entails analyzing the community structure of each mode independently and then combining the community solutions in a manner that maximizes within-community ties [4]–[6]. For example, an affiliation network may be transformed into a person-to-persons network defined by shared committee membership, and a committee-to-committees network defined by shared members [2]. Then any of the many one-mode community detection algorithms [7]–[8] can be leveraged to determine the community structure of each network, and these community solutions can be combined to identify the overall community structure of the entire bipartite network. In particular, this method should outperform the combined approach when the community structures differ between the modes of bipartite networks.

One situation in which the community structures differ between the modes of bipartite networks occurs when there are a different number of communities between the modes. This situation highlights the differences between the combined approach and the dual-projection approach. When using the dual-projection approach, community relevant computations for one mode are not contingent on the structure of the other mode in the same way that they are when using the combined approach. To validate this claim, this paper presents results from network simulations of bipartite networks with different community structures between the modes of the networks. Simulation results indeed support the contention that the dual-projection approach outperforms the combined approach. Below the two approaches are described in more detail. Then, the results from two simulations are reported. The paper concludes with some implications of this work and some potential extensions to consider in the future.

## Communities in Bipartite Networks

The community structure of a bipartite network may be discovered by a variety of means [5]–[6], [9]–[12]. As noted above, commonly the community structure of only one of the modes is analyzed, so the bipartite network is projected into a one-mode network. Suppose we have a binary bipartite network **B** linking artists to teams [13]. The off-diagonal entries in the product 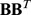 tells us how many teams are shared by each pair of artists, while the diagonal tells us the number of teams to which each artist belongs. This may be analyzed as an unweighted binary projection, or as a weighted projection. The weighted projection approach has been shown to yield superior results [5], but analyzing only one of the modes results in a non-negligible loss of structural information [1].

On the other hand, the community structure of both modes may be of interest. In this case, the community structure of both modes may be discovered simultaneously [6], [9] in a ‘combined’ analysis. One simple approach to doing so is to construct a block off-diagonal meta-matrix [14], [15] which entails the bipartite graph and its transpose. Such a network has the following form
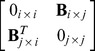
where 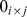 is an all-zero matrix with *i* rows and *j* columns. This structure enables standard one-mode community detection algorithms to provide a solution for both of the modes of a bipartite network simultaneously.

An alternative to the combined approach is to apply the recent dual-projection approach [1]. To do so, take the weighted projection of both modes of a bipartite network, analyze the community structure of both modes separately, and then combine the community partitions in a fashion that maximizes within-community ties. Using the artist-to-teams network, for example, compute the community structure of artists using 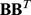, then compute the community structure of teams using 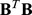, and then maximize over one of the bipartite modularity extensions [5]–[6] to the standard one-mode modularity [4]. This approach also has the benefit of being amenable to all of the standard one-mode community detection algorithms, meaning that no new algorithms or software applications are required for its use. Below I discuss why this strategy is preferred to the combined approach that is discussed above.

The combined approach to identifying communities in networked systems has two related shortcomings. First, all information about the community structure pertaining to one of the modes is indirect. This follows from the simple fact that direct ties between nodes within one of the modes cannot occur within bipartite networks. Second, the community-related computations for the allocation of nodes in one of the modes are affected by the community-related computations for the nodes in the other mode, and this is particularly problematic when the community structure differs between modes. Using the classic Girvan and Newman betweenness-based community detection algorithm [16], for example, all of the geodesics for the nodes in one of the modes require indirect ties through nodes in the other mode when the community structure is discovered using the combined approach. The same is true of other methods such as spectral partitioning [17] or extremal optimization [18]. To validate use of the dual-projection approach relative to the combined approach, below the results of simulated networks with an unequal number of communities between the modes are analyzed using both approaches.

## Test of the Method

To test the performance of the dual-projection approach relative to the combined approach I have applied both of them to a large set of synthetic graphs. An R script is available on the author’s website to replicate the results of the simulations reported in this paper (http://sites.google.com/site/melamedpubssupplementalfiles/). In both cases the community structure was analyzed by maximizing modularity using the walktrap algorithm [19], which places nodes into communities based on neighborhood similarity from short random walks (of 4 steps) and has been shown to be particularly effective at recovering the community structure of large networks. The synthetic bipartite graphs had a density of .125 [16] and within-community ties occurred with a probability of .9. Varying the probability of within-community ties was found to make no substantive impacts on the results of the simulations. Further, the degree distribution for the nodes in the first mode was constrained to be fixed, but the degree of the nodes in the second was not.

Two aspects of the synthetic graphs were manipulated. First, whether there was an unequal number of communities in the two modes was manipulated. Half of the graphs had three communities in the first mode and two in the second. To accomplish this, the first mode was divided into three equally-sized subsections. In the first subsection, within-community ties occurred within the first half of the nodes in the second mode. In the second subsection, within-community ties occurred within the middle third of the nodes in the second mode. Finally, in the third subsection, within-community ties occurred within the second half of the nodes in the second mode. This structure ensured that the most efficient allocation of nodes to non-overlapping communities entailed three communities in the first mode, and two in the second. The other half of the graphs had three equal-sized communities in both of the modes. This manipulation ensures that any increase in effectiveness for the dual-projection approach when there is an unequal number of communities does not come with a concomitant decrease in effectiveness when there is an equal number of communities in the two modes. Second, the size of the synthetic graphs was manipulated. Half of the networks were of dimensions 60×120 and the other half were of dimensions 600×1200. This manipulation ensures that the results are robust to network size since many social networks are typically relatively small, while many physical networks are relatively large.

For the small networks, I relied on 1,000 realizations of each of the networks with equal and unequal community partitions. For the large networks, I relied on 100 realizations of the networks with equal and unequal community partitions. The large density of the large networks was set to .025. Each simulated network was analyzed using the combined and the dual-projection approach, and the normalized mutual information [20] between the known partition and the discovered partition was retained. Results rely on the average normalized mutual information across all of the simulated networks.

**Figure 1:**
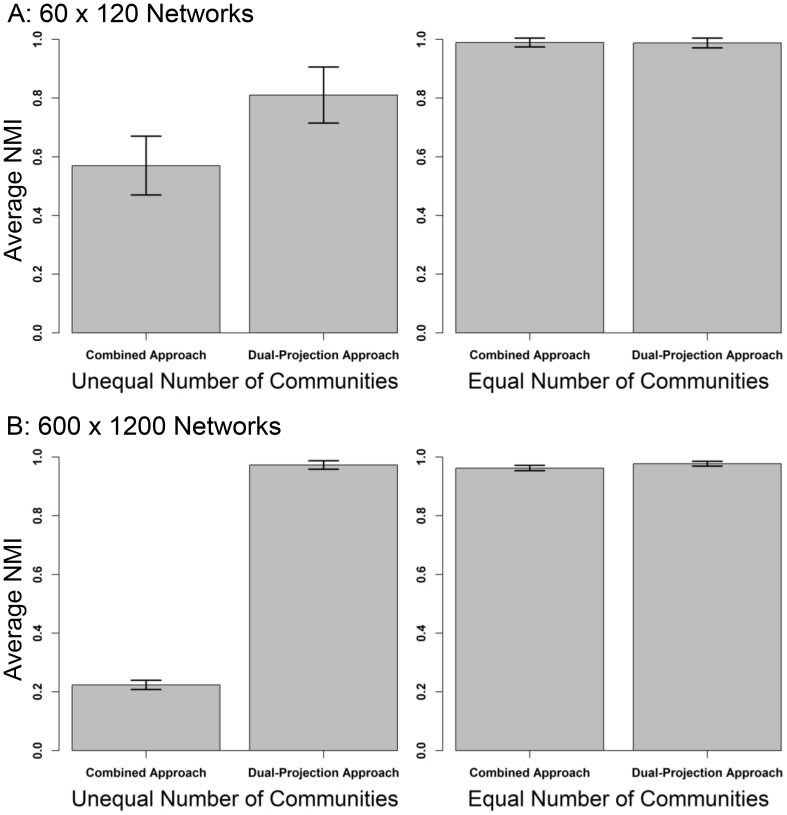
Average normalized mutual information between known and discovered community partitions. Each pair of graphs illustrates the average NMI for the combined approach and the dual-projection approach. The error bars refer to one standard deviation.

Figure 1a presents the results for the smaller networks. When the number of communities is unequal, it is evident that the dual-projection approach outperforms the combined approach (.81 and .57, respectively). At the same time, when the number of communities is equal, there is no difference in the effectiveness of the approaches (both .99). Figure 1b presents the results for the larger networks. When the number of communities is unequal, the discrepancy between the dual-projection approach and the combined approach gets larger: the dual-projection approach is clearly preferred (.97 and .22, respectively). Again, when the number of communities is equal, there is no difference between the approaches (both .99). These results suggest that the dual-projection approach should be preferred to the combined approach: it is more effective when the community structure differs between modes of a bipartite graph, but is just as effective when the community structure between the modes is the same.

While the above results show support for the use of the dual-projection approach when there are an unequal number of communities between the modes in a bipartite network, the two modes differed by only one community. To validate the method when the two modes have a greater disparity in the number of communities, I conducted a second simulation. Relying on the same network size and probability of within-community ties, I generated networks with two communities in the first mode and ten in the second. To accomplish this, the nodes in the first mode were divided into twenty equally-sized subsections. In the first subsection, within-community ties occurred within the first tenth of the nodes in the second mode. In the second subsection, within-community ties occurred within the second tenth of the nodes in the second mode. The third subsection of nodes in the first mode linked the first two subsections by overlapping a thirtieth of the nodes in the second mode with the first and second tenths of the nodes in the second mode. Each of the remaining subsections in the first mode were allocated similarly, except that there was no overlap in the second mode between the tenth and eleventh subsections from the first mode, which ensured that a two community solution to the first mode was the optimal partition.

**Figure 2:**
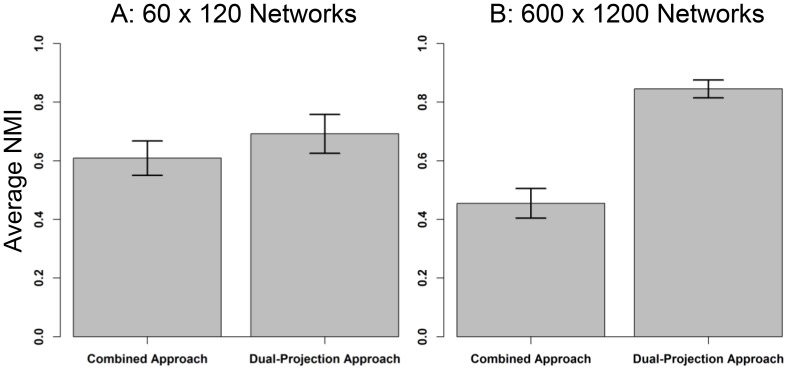
Average normalized mutual information between known and discovered community partitions. The pair of graphs illustrates the average NMI for the combined approach and the dual-projection approach. All networks had two communities in the first mode and ten in the second. The error bars refer to one standard deviation.

The simulated networks, as described above, were again analyzed using both the combined and dual-projection approaches. One thousand small networks and 100 large networks were analyzed. Figure 2 presents the results. Again, the dual-projection approach outperforms the combined approach. The difference in the small networks is relatively small (.69 and .61, respectively), but the sample is quite large. As was observed in the first simulation, in the large networks the discrepancy becomes quite a bit larger (.85 and .45, respectively). Thus the dual-projection approach remains preferred with more unequal community structures between the modes of a bipartite network.

## Conclusions

This paper illustrated that the dual-projection approach to detecting communities in bipartite networks should be preferred to the combined approach. In particular, sub-optimal community solutions may be identified when using the combined approach. As such, the results are more likely to misidentify sub-structures of bipartite networks, which in turn, may yield mistakes about the function and topology of the network. With respect to the dual-projection approach, all of the standard one-mode algorithms for community detection can be applied to discover the community structure of bipartite networks. That is to say, the increased accuracy of the dual-projection approach does not come with a corresponding loss of generality. Of particular relevance, the dual-projection approach has been shown to outperform the combined approach to detecting communities in bipartite networks when the community structure differs between modes of the network.

While it was assumed for the sake of simplicity up to this point that all of the nodes in graphs should be partitioned into unique communities, the dual-projection approach does not require this assumption. The dual-projection approach to partitioning bipartite graphs is particularly flexible and can be applied in conjunction with the evolving literatures on overlapping and hierarchical community structures [21]–[28]. Depending on the substance of the research question, it may be the case that one of the modes should be partitioned into non-overlapping communities, while the other mode should incorporate overlapping communities. For example, in a network of committee assignments it may be reasonable to assume that people may be allocated into overlapping communities while committees may not because they are often developed to account for discrete phenomenon. Likewise, one of the modes may entail hierarchical communities, while the other entails overlapping communities.

Another factor that has been ignored up to this point is that many networked systems have more than two modes. Some literature deals with community structures in such networks [9], [29]. The dual-projection approach can easily be generalized to *k*-partite graphs. The main insight here is that for any *k*, there are *k* choose two bipartite graphs that can be analyzed using the dual-projection approach. Of course, analysts can assume any form of community structure associated with the various modes embedded within the larger *k*-partite graphs.

In summary, it has been illustrated that the dual-projection approach to community detection in bipartite graphs is preferred to the combined approach. The dual-projection approach determines the community structure of each mode of bipartite networks separately while the combined approach determines them simultaneously. Results from the analysis of synthetic networks showed that the dual-projection approach outperforms the combined approach when the number of communities in the two modes of bipartite networks are unequal. At the same time, the dual-projection approach performs just as well when the number of communities in the two modes is equal. The dual-projection approach to community identification is flexible and warrants further investigation.
